# The use of POTTER (Predictive Optimal Trees in Emergency Surgery Risk) calculator to predict mortality and complications in patients submitted to Emergency Surgery

**DOI:** 10.1590/0100-6991e-20233624-en

**Published:** 2023-11-18

**Authors:** Marcelo Augusto Fontenelle Ribeiro, Rafaela Smaniotto, Anthony Gebran, Jefferson Proano Zamudio, Shahin Mohseni, José Mauro da Silva Rodrigues, Haytham Kaafarani

**Affiliations:** 1 - Pontificia Universidade Católica de São Paulo Campus Sorocaba, Disciplina de Cirurgia Geral - Sorocaba - SP - Brasil; 2 - Sheikh Shakhbout Medical City, Division of Trauma, Critical Care and Acute Care Surgery - Abu Dhabi - AD -Emirados Árabes Unidos; 3 - Khalifa University, Department of Surgery - Abu Dhabi - AD - Emirados Árabes Unidos; 4 - University of Pittsburgh Medical Center, Department of Surgery - Pittsburgh - PA - Estados Unidos; 5 - Harvard Medical School, Trauma, Emergency Surgery and Surgical Critical Care, Massachusetts General Hospital - Boston - MA - Estados Unidos

**Keywords:** Artificial Intelligence, Complication, Morbidity, Mortality, Risk Factors, Inteligência Artificial, Complicações Pós-Operatórias, Mortalidade, Morbidade, Fatores de Risco

## Abstract

**Introduction:**

the ability of the care team to reliably predict postoperative risk is essential for improvements in surgical decision-making, patient and family counseling, and resource allocation in hospitals. The Artificial Intelligence (AI)-powered POTTER (Predictive Optimal Trees in Emergency Surgery Risk) calculator represents a user-friendly interface and has since been downloaded in its iPhone and Android format by thousands of surgeons worldwide. It was originally developed to be used in non-traumatic emergency surgery patients. However, Potter has not been validated outside the US yet. In this study, we aimed to validate the POTTER calculator in a Brazilian academic hospital.

**Methods:**

mortality and morbidity were analyzed using the POTTER calculator in both trauma and non-trauma emergency surgery patients submitted to surgical treatment between November 2020 and July 2021. A total of 194 patients were prospectively included in this analysis.

**Results:**

regarding the presence of comorbidities, about 20% of the population were diabetics and 30% were smokers. A total of 47.4% of the patients had hypertensive prednisone. After the analysis of the results, we identified an adequate capability to predict 30-day mortality and morbidity for this group of patients.

**Conclusion:**

the POTTER calculator presented excellent performance in predicting both morbidity and mortality in the studied population, representing an important tool for surgical teams to define risks, benefits, and outcomes for the emergency surgery population.

## RESUMO

**Introdução:**

a capacidade da equipe de atendimento de prever de forma confiável o risco pós-operatório é essencial para melhorar a tomada de decisões cirúrgicas, o aconselhamento ao paciente e à família e a alocação de recursos nos hospitais. A calculadora POTTER (Predictive Optimal Trees in Emergency Surgery Risk), alimentada por inteligência artificial (IA) e com uma interface amigável, foi baixada em seu formato para iPhone e Android por milhares de cirurgiões em todo o mundo e foi originalmente desenvolvida para ser usada em pacientes de cirurgia de emergência não traumática. No entanto, a POTTER ainda não foi validada fora dos EUA. Neste estudo, nosso objetivo foi validar a calculadora POTTER em um hospital acadêmico brasileiro.

**Métodos:**

a mortalidade e a morbidade foram analisadas usando a calculadora POTTER em pacientes de cirurgia de emergência com e sem trauma submetidos a tratamento cirúrgico entre novembro de 2020 e julho de 2021. Um total de 194 pacientes foi incluído prospectivamente nessa análise.

**Resultados:**

Em relação à presença de comorbidades, cerca de 20% da população era diabética e 30%, fumante. Um total de 47,4% dos pacientes eram hipertensos antes da admissão. Após a análise dos resultados, identificamos uma capacidade adequada de prever a mortalidade e a morbidade em 30 dias para esse grupo de pacientes.

**Conclusão:**

a calculadora POTTER apresentou um excelente desempenho para prever a morbidade e a mortalidade na população estudada, representando uma ferramenta importante para as equipes cirúrgicas definirem riscos, benefícios e resultados para a população de cirurgia de emergência.

## INTRODUCTION

Emergency general surgeries (EGS) are a public health problem, have increased in recent decades, and continue to represent a large portion of non-elective surgical activities worldwide, including Brazil. Currently, Brazil lacks reliable data on the number of emergency surgery admissions and lacks a standardized methodology for surgical risk analysis.

When compared to similar elective surgeries, emergency surgeries have been shown to have a much higher risk (up to 8 folds higher) of complications and postoperative mortality^1^ ^-^ ^3^. As such, the ability of the care team to reliably predict postoperative risk is essential for improvements in surgical decision-making, patient and family counseling, and resource allocation in hospitals^4^. Many risk stratification models currently exist, including but not limited to: American Society of Anesthesiologists (ASA) classification^5^, Elixhauser Comorbidity Index^6^, Charlson Comorbidity Index^7^, Surgical Risk Calculator (ACS-SRC)^8^ and the Emergency Surgery Score (ESS)^11^ ^-^ ^13^. Except ESS, these models were derived mostly from and for elective surgery patients, and their accuracy and use for emergency surgery patients remain in question^9^ ^,^ ^10^. When ESS was developed, it was suggested to be a better predictive model for emergency surgery patients^11^ ^-^ ^13^.

Nonetheless, all these aforementioned risk stratification models are based on the idea that the variables used to calculate risk interact in a linear and additive manner. However, medical reality suggests that patients’ comorbidities and disease markers interact in a complex, non-linear way and that some variables may gain or lose strength depending on the presence or absence of other variables^14^.

In this context, the Artificial Intelligence (AI)-powered POTTER (Predictive Optimal Trees in Emergency Surgery Risk)^4^ calculator was recently developed using nearly 400 thousand emergency surgery patients and uses a non-linear, novel, and transparent machine learning methodology to estimate the risk of postoperative mortality and complications. The POTTER user-friendly interface has since been downloaded in its iPhone and Android format by thousands of surgeons worldwide.

POTTER derivation and validation have been previously described^4^. Briefly, all patients who underwent emergency surgery in the ACS NSQIP database (2007-2013) were used to train Optimal Classification Trees (OCT) for the development and validation of the POTTER calculator. OCTs are novel, interpretable, machine learning (ML)-based methodologies that follow a sequence of splits (nodes) on key variables to make a final prediction. POTTER effectively predicts the postoperative outcomes of emergency surgery patients and outperforms all other risk calculators in the field (the c-statistic for predicting mortality in EGS (Emergency General Surgery) patients is 0.92).

However, POTTER has not been validated outside the US yet. In this study, we aimed to validate the POTTER calculator in Brazil not only for emergency surgery patients but also expand its use for trauma patients, aiming to evaluate its capacity to predict the same variables as for emergency surgery cases. 

## METHODS

### Patient population

This validation study was carried out in a southeastern Brazilian city, Sorocaba, with a population estimated at around one million people. All patients over 18 years old who were admitted to the General Surgery service at Conjunto Hospitalar de Sorocaba, between November 2020 and July 2021, and were submitted to any kind of emergency surgery procedure were included and are presented in table X as per the admission diagnosis according to the medical records. Both trauma and non-trauma emergency surgery patients were included. Although the POTTER application was developed using artificial intelligence using data from patients undergoing emergency surgery who were not trauma victims, we opted to add trauma cases undergoing surgical treatment to evaluate if the ability to assess the risks of complications and deaths would be similar to cases of patients who were not trauma victims. The IRB review committee at the Conjunto Hospitalar de Sorocaba reviewed and approved this study - IRB register number 5.013.427.

### Data variables and POTTER prediction

Medical records were systematically reviewed, and the following information’s were collected according to the requirements of the app based on artificial intelligence database to calculate the complications and mortality rates: Age, laboratory values (hematocrit, white blood cell count, platelet, sodium, potassium, blood urea nitrogen, creatinine, albumin, bilirubin, serum glutamic-oxaloacetic transaminase, alkaline phosphatase, partial thromboplastin time, international normalized ratio), comorbidities (COPD, diabetes, smoking, hypertension, acute renal failure, ascites, congestive renal failure, cancer bleeding disorders), intensive care unit (ICU) admission, and complications (fistula, septic shock, aponeurosis dehiscence, pulmonary thromboembolism, anastomosis dehiscence, wound infection, intracavitary abscess, and evisceration), The ACS_NSQIP definitions were used in data collection. Using the collected data, the POTTER predictions of 30-day mortality and 30-day morbidity were calculated for each patient using the existing algorithms and phone application. The primary outcome was POTTER’s accuracy in predicting 30-day mortality. The secondary end point was to establish POTTER’s accuracy in predicting overall 30-day morbidity. Analyses were performed for the overall cohort as well as the non-trauma subpopulation.

### Statistical analysis

The area under the receiver operator characteristic curve (AUC), or c-statistic measure, was used to assess the relationship between POTTER’s predictions and the outcomes of interest. STATA Software, version 15.1 was used for statistical analysis (Stata Corp).

## RESULTS

A total of 194 patients composed of the ESTG (Emergency Surgery and Trauma Group) were included in this study; out of these, 169 were emergency surgery patients with no trauma, and 25 patients were included due to trauma aiming to understand the applicability of this predicting model to trauma patients. The demographic characteristics of the patients and clinical pre-operative comorbidities are presented in Tables 2 and 3. There was a predominance of mid-age males (112 patients) with good functional status prior to admission. Regarding the presence of comorbities, about 20% of the population were diabetics and 30% were smokers. A total of 47.4% of the patients were hypertensive pre-admission. 

[Table t1]

**Table 1 t1:** Diagnosis at admission for patients submitted to surgical procedure.

Diagnosis at admission	Number of cases
Acute appendicitis	50
Acute cholecystitis	21
Perforating acute abdomen	21
Obstructive acute abdomen	19
Left Colon & sigmoid cancer	13
Stab wound	11
Blunt abdominal trauma	9
Diverticulitis	6
Vascular acute abdomen	6
Gunshot wound	5
Liver abscess	4
Perianal abscess	4
Cervical abscess	3
Gallbladder cancer	2
Genital abscess	2
Incarcerated hernia	2
Fournier gangrene	1
Cecum perforation	1
Chagasic megacolon	1
Advanced gastric adenocarcinoma	1
Diagnosis at admission	Number of cases
Gynecological inflammatory acute abdomen	1
Bladder tumor	1
Cholecystoduodenal fistula	1
Lower gastrointestinal bleeding	1
Pancreatic head neoplasm with cholangitis	1
Anastomotic fistula	1
Perforation due to Crohn's disease	1
Proximal jejunum neoplasm	1
Cecum cancer	2
Complicated ulcerative colitis	1
Enterocutaneous fistula	1
Total	194

**Table 2:** Demographic characteristics of the population.

Variables	Overall Cohort (n=194)
Age (median)	53 (18-84)
Q_1_	32
Q_2_	53
Q_3_	66
Q_4_	84
Gender, number(%)	
Male	112 (57.7%)
Female	82 (42.3%)
Race, number (%)	
White	102 (52.57%)
Black or mixed-race	90 (46.39%)
Asian	2 (1.03%)
Functional Status	
Independent	168 (86.59%)
Partially dependent	22 (11.34%)
Totally dependent	4 (2.06%)

**Table 3:** Pre- operative comorbidities in the study population.

Pre-operative comorbidities	total number (%)
Diabetes	42(21.64%)
Smoker	58(29.89%)
Dyspnea	
At rest	5 (2.57%)
Moderate Exertion	11 (5.67%)
History of severe COPD	20 (10.30%)
Ascites	7 (3.60%)
Congestive Heart Failure	4 (2.06%)
Hypertension requiring medication	92 (47.42%)
Acute renal Failure	0
Currently on dialysis	0
Disseminated cancer	9 (4.63%)
Open wound / Wound infection	6 (3.09%)
Steroid use for chronic condition	13(6.70%)
Bleeding disorders	8 (4.12%)
Preoperative transfusion	17 (8.76%)
Systemic Sepsis	
Sepsis	54 (27.83%)
Septic Shock	2 (1.03%)
Patients who died	20 (10.30%)
Patients with any complication	44 (22.68%)

COPD - Chronic obstructive pulmonary disease

Regarding the presence of sepsis, approximately 28% of the patients presented with this condition during admission. The number of complications were 22.6% and 10% of the patients died. 

**Figure 1:**
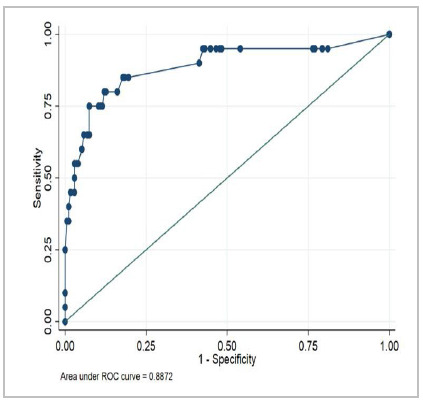
Receiver Operating Characteristic (ROC) Curve for 30-day Mortality (ESTG).

**Figure 2:**
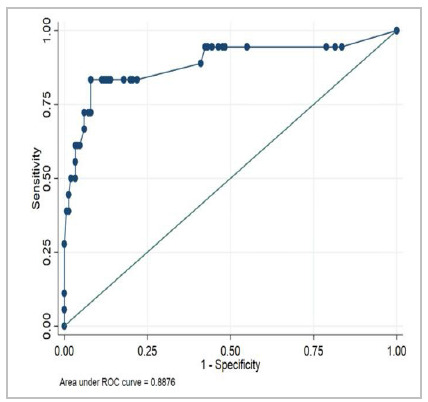
Receiver Operating Characteristic (ROC) Curve for 30-day Mortality (ESG).

After analyzing the data regarding mortality in the Emergency Surgery and Trauma group (ESTG) and in the Emergency Surgery group (ESG), we can identify an adequate capability to predict 30-day mortality (p<0.001), as demonstrated in Table 4 and Figures 1 and 2.

**Table 4:** Predictive Performance of POTTER for 30-day Mortality ESTG and ESG groups.

				95% Confidence Interval	
ESTG group	Number of Patients	Area Under the ROC	p-value	LCL	UCL
	194	0.8872	<0.001	0.79037	0.98406
ESG group	169	0.8876	<0.001	0.77934	0.99586

**Figure 3:**
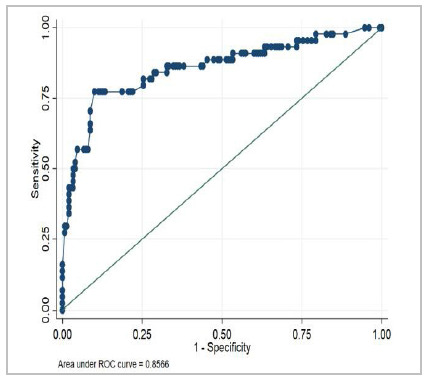
Receiver Operating Characteristic (ROC) Curve for 30-day Morbidity (ESTG).

**Figure 4:**
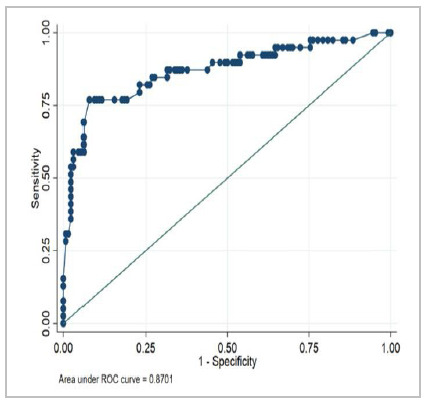
Receiver Operating Characteristic (ROC) Curve for 30-day Combined Morbidity (ESG).

The performance of POTTER to predict morbidity was evaluated for both ESTG and ESG according to Tables 5 and Figures 3 and 4, and in all the groups we were able to demonstrate a p<0.001, proving the applicability of the POTTER.

**Table 5:** Predictive Performance of POTTER for 30-day Combined Morbidity ESTG and ESG groups.

				95% Confidence Interval	
ESTG group	Number of Observations	Area Under the ROC	p-value	LCL	UCL
	194	0.8566	<0.001	0.78351	0.92968
ESG group	169	0.8701	<0.001	0.79534	0.9449

## DISCUSSION

In this study, we conducted a prospective validation of the artificial intelligence tool POTTER in a mixed cohort of Brazilian emergency surgery and trauma patients. The algorithm showed high predictive accuracy for mortality and morbidity. POTTER was trained on the National Surgical Quality Improvement Program (NSQIP) database, which has existed since 2005 and contains pre-operative, operative, and post-operative data on millions of patients who underwent emergent or non-emergent surgical intervention^4^. It is without question the largest and best-validated surgical database in the world. There are two other notable tools derived from this dataset that have been examined in emergency surgery: the NSQIP Surgical Risk Calculator (SRC) and the ESS1^15^ but POTTER has clear advantages over these two instruments. 

First, in contrast to the official NSQIP surgical risk calculator, which has been shown to perform poorly in emergency general surgery^9^, POTTER was purposefully designed for use in the emergency setting and considered only variables available in the pre-operative setting. It has been validated in emergency laparotomies, and in the elderly^2^ ^,^ ^3^ which highlights its robustness in these two sub-populations of ES. Our study is one of the first to show prospectively that POTTER has high predictive accuracy in an external cohort.

Second, when compared to ESS, which was also designed to be deployed in surgical emergencies, POTTER not only outperformed ESS in the original development and validation cohorts, but due to its dynamic, non-linear nature, it represents the various physiology-comorbidity combinations that occur in the clinical realm more faithfully. Furthermore, when ESS was prospectively validated, it achieved a c-static of 0.84 for the prediction of 30-day mortality and 0.74 for 30-morbidity^4^; in our validation study, POTTER achieved a c-statistic of 0.89 for mortality and 0.86 for morbidity, which further confirms POTTER’s superiority as a predictive model.

Our study is the first international validation of this artificial intelligence tool developed in the United States. Furthermore, due to our inclusion of both trauma and non-trauma patients requiring emergent surgical intervention, the results provide evidence of its usefulness and applicability to a broader population than that previously described. Together with its simple and user-friendly interface in the form of a smartphone application, makes POTTER very easy to deploy at the patient’s bedside in the emergency department^5^, which makes it particularly useful in settings where access to a comprehensive electronic health record or similar interface is limited, especially in places like public hospitals in developing countries where internet access is still very limited, making this tool practical and accessible.

This study should be interpreted with the following limitations in mind: First, patients were recruited in a single institution, and sample size is limited. Second, there are some clinically relevant variables (other comorbidities, arterial blood gases, imaging results, etc.) that are not available on NSQIP and therefore not considered by POTTER and could affect the risk prediction. Finally, due to the limited number of events, POTTER’s performance in individual complications could not be assessed. Despite the fact that the POTTER application was developed for use in emergency surgeries unrelated to trauma, we were able, albeit with a small and limited sample, to demonstrate that the ability to predict complications and mortality was adequate even when trauma cases were added, opening up perspectives for new tools using artificial intelligence to be developed specifically for this population and thus helping medical teams identify patients with a greater potential risk of both complications and death after trauma.

In conclusion, we successfully validated the Predictive Optimal Trees in Emergency Surgery Risk tool in a Brazilian emergency surgery population. This predictive model showed high predictive accuracy for the outcomes of 30-day mortality and 30-day morbidity. This user-friendly algorithm can provide a reliable prediction at the patient’s bedside in a rapid fashion. Further studies are needed to examine how its use impacts outcomes in different health systems around the world.
